# Associations between early-life adversity, coping strategies, and adult mental health, brain, and cognition

**DOI:** 10.1038/s41598-026-42435-w

**Published:** 2026-03-04

**Authors:** Morgane Künzi, D. A. Gheorghe, J. Lian, S. Bauermeister

**Affiliations:** 1 Dementias Platform UK, Department of Psychiatry, University of Oxford, Warneford Hospital, OX3 7JX Oxford, UK; 2 Department of Experimental and Theoretical Neuroscience, Transylvanian Institute of Neuroscience, Cluj-Napoca, Romania

**Keywords:** ACE, Executive function, Stress-related behaviours, Neuroimaging, Lifestyle, Personality, Diseases, Health care, Neuroscience, Psychology, Psychology, Risk factors

## Abstract

Early adversity is associated with later mental health, brain, and cognitive outcomes, but the pathways are complex and may involve coping strategies and individual vulnerabilities. We investigated associations between early adversity, coping strategies, neuroticism, and adult mental health, cognition, and global brain volumes. Path analysis was applied to behavioural and imaging data from the UK Biobank dataset (*N* = 472,450, *Mdn*_*age*_ = 58, *SD*_*age*_ = 8.03, 54.46% of women). All assessed early adverse experiences were associated with greater anxiety symptoms, while all except physical neglect were associated with increased depressive symptoms. Physical neglect was the only adversity associated with poorer cognitive performance, and no adversity showed a direct association with global grey- or white-matter or cerebrospinal fluid volumes. Several indirect pathways were observed: specific coping strategies and neuroticism significantly mediated links between early adversity and adult mental health, cognition, and cerebrospinal fluid volume. These findings are consistent with prior work linking early adversity to adult mental health and brain measures, and highlight coping behaviours and neuroticism as mediating factors. Strengthening adaptive coping may mitigate some detrimental associations, but causal inference is limited by the cross-sectional study design.

**Supplementary Information:**

The online version contains supplementary material available at 10.1038/s41598-026-42435-w.

## Introduction

Early adversity is defined as “highly stressful, and potentially traumatic, events or situations”^1^. A substantial body of evidence links early adverse experiences to poorer mental health outcomes, altered brain structure, and poorer cognition later in life^2–10^. However, these associations are heterogeneous and vary according to the adversity characteristics such as type, severity, duration, chronicity, developmental period, and co-occurrence with other adverse experiences^2,9,11^.

Several non-mutually exclusive pathways have been proposed to explain how early adversity may influence later mental health, brain, and cognitive outcomes. These include stress-response systems, allostatic load (stress-related “wear and tear” on the body), structural and functional brain disruptions, personality traits, and coping strategies used for adaptation (e.g.,^2,12–18^). Stress is an important component in these pathways, where stressors experienced during periods of brain development and, therefore, heightened vulnerability potentially exert both direct and indirect effects on brain structure, function, and cognition^15,16,19,20^.

Exposure to stressors such as early adversity elicits psychological and behavioural responses aimed at adaptation. Coping is defined by Lazarus and Folkman^21^ as “constantly changing cognitive and behavioural efforts to manage specific external and/or internal demands that are appraised as taxing or exceeding the resources of the person”. Importantly, coping represents the effort to manage stress rather than the effectiveness or adequacy of the response^21^. In empirical research, behaviours conceptualised as coping may range from adaptive responses (e.g., social engagement) to maladaptive responses (e.g., substance use or self-harm-related thoughts). While some of these behaviours may not constitute deliberate coping strategies in a strict theoretical sense, they nevertheless represent meaningful behavioural responses to stress exposure and may play a role in shaping long-term mental health and cognitive outcomes.

In addition to behavioural responses, personality traits such as neuroticism have been implicated as potential vulnerability factors linking early adversity to later psychopathology. Neuroticism is associated with heightened stress sensitivity and negative emotionality and has been consistently linked to anxiety and depressive disorders^13,22^. Evidence suggests that neuroticism predicts poorer adult emotional health in the context of childhood adversity^23^.

Despite extensive research documenting associations between early adversity and adult outcomes, it is unclear the extent to which coping-related behaviours and personality jointly account for links between early adversity, mental health, brain structure, and cognition. Many studies focus on isolated outcomes or single pathways, limiting understanding of how these factors interact within a broader psychosocial and neurobiological context.

Our study addresses this gap by examining associations between early adverse experiences, coping strategies, neuroticism, and adult mental health, brain volumes, and cognition within a single integrative framework. Using path analysis in a large population-based cohort, we investigate both direct associations and indirect pathways linking early adversity to adult outcomes via coping-related strategies and neuroticism. By adopting this approach, the study aims to clarify patterns of association across multiple domains and contribute to a more nuanced understanding of potential pathways linking early adversity to later-life mental health, brain, and cognition.

## Methods

### Participants

Data came from the UK Biobank (*N* = 472,450, *Mdn*_*age*_ = 58, *SD*_*age*_ = 8.03, 54.46% of women). Early adversity was assessed in 2016 using an online questionnaire. For coping strategies, the number of social activities was collected in 2014+, while data on “ever addicted to any substance or behavior”, “ever contemplated self-harm”, and “having been in a confiding relationship as an adult” were collected in 2016. For the outcomes, mental health (i.e., depressive and anxiety symptoms) was collected in 2016, cognition was assessed in 2019+, and brain imaging data were collected in 2019+ (see Tables 1 and 2).

**Table 1:** Frequency table of early adversity items.

Early adversity	Yes	No	Missing
Physical neglect	24,193 (5.12%)	126,997 (26.88%)	321,260 (68.00%)
Sexual abuse	12,873 (2.72%)	137,597 (29.12%)	321,980 (68.15%)
Emotional neglect	72,263 (15.30%)	79,352 (16.80%)	320,835 (67.91%)
Physical abuse	28,097 (5.95%)	123,773 (26.20%)	320,580 (67.85%)
Emotional abuse	23,183 (4.91%)	128,590 (27.22%)	320,677 (67.88%)

**Table 2:** Descriptive statistics of outcomes and control variables in the UK Biobank.

Variables	*n*	Mdn	SD	Min	Max
Addition	150,484	0	0.24	0	1
Social activities	54,987	1	0.68	1	5
Self-harm contemplation	151,585	0	0.35	0	1
Confiding relationship	148,282	1	0.28	0	1
Depressive symptoms without/with transformation	149,364	2/1.10	3.67/0.83	0/0	27/3.33
Anxiety symptoms without/with transformation	150,050	0/0	3.39/0.85	0/0	21/3.09
Fluid intelligence	6400	7	2.01	0	13
TMTB without/with transformation	6570	496/6.21	255.56/0.36	197/5.28	4413/8.39
Grey matter volume (V_GM2)	4633	613,566	54815.31	457,042	779,237
White matter volume (V_WM2)	4631	537,868	60016.94	369,727	723,665
Cerebrospinal fluid volume	4576	33513.95	15182.27	9329.02	88076.2
Neuroticism	382,555	4	3.26	0	12
Age	472,450	58	8.03	40	73
Education	468,495	17	2.73	5	35

*n* = Number of respondents, *Mdn* = Median, SD = Standard Deviation, *Min* = Minimum range value, and *Max* = Maximum range value.

## Materials

### Early adversities

Early adversity was measured using the Childhood Trauma Questionnaire (CTS-5), including: emotional abuse (“I felt that someone in my family hated me”), sexual abuse (“someone molested me sexually”), emotional neglect (“I felt loved a child”), physical abuse (“People in my family hit me so hard that it left me with bruises or marks”), and physical neglect (“There was someone to take me to the doctor if I needed it”). The items were dichotomised following previous procedures^5^ (Table 1).

### Coping strategies

#### Addiction

Addiction was assessed with the item “ever addicted to any substance or behaviour”. The item was coded 0 = no and 1 = yes, the other possible answers (i.e., prefer not to answer and do not know) were coded as missing values.

#### Social activities

Social activities were assessed in 2014 + with the item: “Which of the following do you attend once a week or more often”. Five possible activities were provided, with multiple answers allowed: sports club or gym, pub or social club, religious group, adult education class, other activity. “None of the above” and “Prefer not to answer” responses were recoded as missing values. Based on the answers provided, a sum score of the social activities undertaken was computed, ranging from 1 to 5.

#### Self-harm contemplation

Self-harm contemplation was assessed using the item “Have you contemplated harming yourself (for example by cutting, biting, hitting yourself, or taking an overdose)?”. The item was coded binary regardless of the number of times. “Prefer not to answer” responses were recoded as missing values.

#### Confiding relationship

Being in a confiding relationship was assessed using the item: “Since I was sixteen… I have been in a confiding relationship”. This item was dichotomised in the same manner as the early adversity items.

### Outcomes

#### Depressive symptoms

Depressive symptoms were measured using the Patient Health Questionnaire-9 questions (PHQ-9). Participants responded on a scale from 0 (not at all) to 3 (nearly every day) to indicate whether they had been bothered by a list of 9 problems in the past 2 weeks. Based on the answers provided, a sum score was computed and a LN + 1 transformation was applied given the right skewness in the data.

#### Anxiety

Anxiety was measured using the Generalized Anxiety Disorder-7 (GAD-7). Participants rated how often they had been bothered by 7 problems in the past 2 weeks, on a scale from 0 (not at all) to 3 (nearly every day). Based on the answers, a sum score was computed and a LN + 1 transformation was applied given the right skewness in the data.

#### Fluid intelligence

Fluid intelligence was assessed using 13 items across different domains, which participants were required to complete within a 2-minute time limit. The UK Biobank-derived score (from 0 to 13) was used for analysis (for more information, see the UK Biobank website: https://biobank.ndph.ox.ac.uk/showcase/field.cgi?id=20016).

#### Trail making Test B

The Trail Making Test Part B (TMTB) measured time in seconds to correctly connect the numbers 1 to 13 and the letters A to L in ascending and alphabetic order, alternating between numbers and letters (1-A, 2-B, 3-C,.,12-L-13). A log transformation was applied as the data were not normally distributed (see^24^ for a similar procedure).

#### Brain imaging

Total grey matter, white matter, and cerebrospinal fluid (CSF) volumes were UK Biobank-derived from T1-weighted structural MRI scans. Scanning was performed on Siemens Skyra 3T scanners using harmonised protocols. Image processing and extraction of brain volume metrics were conducted using the UK Biobank imaging pipeline, with tissue-type segmentation performed using FAST - FMRIB’s Automated Segmentation Tool^25^. Full details of the scanning and analysis procedures are available on the UK Biobank website: http://biobank.ndph.ox.ac.uk/showcase/label.cgi?id=100 and have been documented previously^26^.

### Control variables

#### Neuroticism

Neuroticism was assessed using 12 neurotic behaviour items. A composite score was derived and provided by the UK Biobank (for more information, see the UK Biobank website: https://biobank.ndph.ox.ac.uk/showcase/field.cgi?id=20127).

#### Age

Age at recruitment was used.

#### Education

The number of years of education was used, and the missing data were imputed using the variable “qualifications achieved”.

### Statistical analysis

STATA V. 18.0 was used for data processing and path modelling (StataCorp, College Station, TX, USA). All non-dichotomous variables were centred. To account for ethnicity as a confounding variable and due to the unbalanced data in the different subcategories, only individuals of white ethnicity were included in the analysis.

The path modelling was defined as follows: all early adversities (physical neglect, sexual abuse, emotional neglect, physical abuse, emotional abuse) predicted the potential mediators (have/had any addiction, the number of social activities undertaken, self-harm contemplation, having been in a confiding relationship, neuroticism score) and the outcomes (anxiety, and depressive symptoms, fluid intelligence score, time completion at TMTB, the volume of grey and white matter as well as the CSF volumes). The potential mediators (if have/had any addiction, the number of social activities undertaken, self-harm contemplation, having been in a confiding relationship, neuroticism score) predicted the outcomes (anxiety and depressive symptoms, fluid intelligence score, time completion at TMTB, the volume of grey and white matter, as well as the volume of CSF). Age, sex, and education predicted the potential mediators (if have/had any addiction, the number of social activities undertaken, self-harm contemplation, having been in a confiding relationship, neuroticism score) and the outcomes (anxiety, and depressive symptoms, fluid intelligence score, time completion at TMTB, the volume of grey and white matter as well as the volume of CSF). All the exogenous variables (early adversities, age, sex, and education) covaried. The error terms of all the potential mediators (if have/had any addiction, the number of social activities undertaken, self-harm contemplation, having been in a confiding relationship, and neuroticism score) were also allowed to covary between them.

**Fig. 1:**
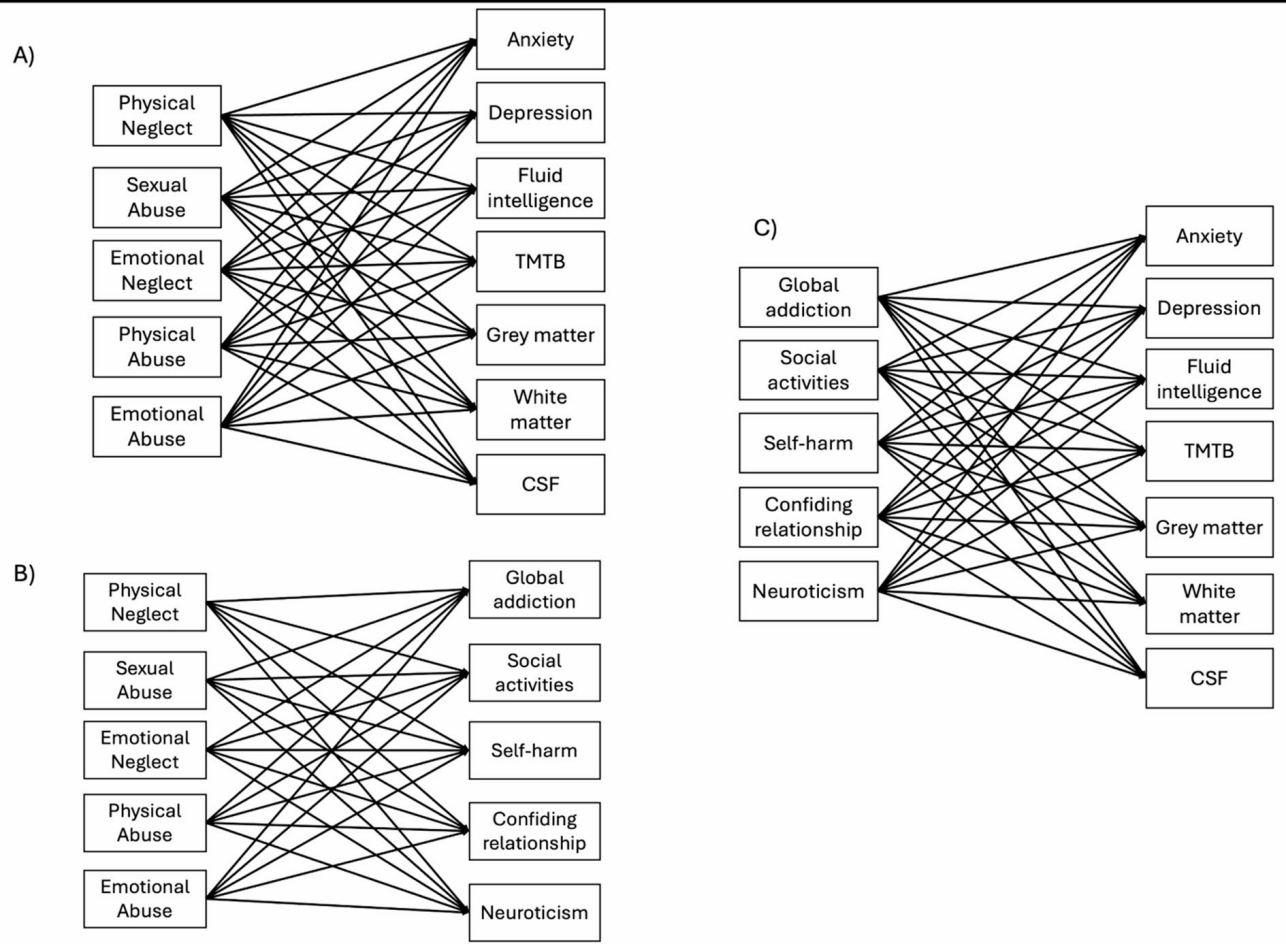
Illustration of the complete model fitted decomposed into 3 parts. For clarity purposes, the model was decomposed into 3 parts, and the control variables and covariances between variables were not drawn. (**A**) illustrates the paths between early adversities and the outcomes of interest, (**B**) illustrates the paths between early adversities and the potential mediators, and (**C**) illustrates the paths between the potential mediators and the outcomes of interest.

For the outcomes, the error terms of the mental health variables (anxiety and depressive symptoms) were allowed to covary, as were the error terms of the cognitive variables (fluid intelligence score and TMTB completion time). Finally, the error terms of the brain variables (volume of grey and white matter and volume of CSF) covaried (Fig. 1). The path analysis was performed with α = 0.001 to limit type I errors.

## Results

The model fit was good: CFI = 0.998, and RMSEA = 0.006^27,28^. As indicated by the variance inflation factor (VIF) ≤ 1.22, there was no multicollinearity between the exogenous variables in the model (i.e., physical neglect, sexual abuse, emotional neglect, physical abuse, emotional abuse, age, sex, and education).

### Anxiety

Physical neglect, sexual abuse, emotional neglect, physical abuse, and emotional abuse in childhood significantly predicted more anxiety symptoms (see Table 3 and Supplementary Figure A).

**Table 3:** Standardised coefficients of the model with early adversity as predictor.

Outcomes	Early adversity					Control variables		
	Physical neglect	Sexual abuse	Emotional neglect	Physical abuse	Emotional abuse	Age	Sex	Education
Addiction	0.006	0.049**	0.037**	0.022**	0.076**	− 0.058*	0.051**	0.014**
Social activities	− 0.007	0.007	− 0.025**	0.008	− 0.008	0.078**	− 0.074*	0.119**
Self-harm contemplation	0.004	0.090**	0.076**	0.053**	0.137**	− 0.125*	− 0.058*	0.022**
Confiding relationship	− 0.099**	0.022**	− 0.013**	0.013**	0.011**	− 0.004	0.010**	0.123**
Neuroticism	0.011**	0.031**	0.103**	0.007	0.110**	− 0.107*	− 0.134*	− 0.077**
Depressive symptoms	0.005	0.035**	0.036**	0.032**	0.067**	− 0.079*	− 0.044*	− 0.023**
Anxiety symptoms	0.024**	0.020**	0.022**	0.016**	0.054**	− 0.077*	− 0.048*	0.005
Fluid intelligence	− 0.106**	0.008	0.014	0.002	− 0.013	− 0.094*	0.030	0.293**
Trail Making Test B	0.081**	− 0.016	0.006	0.012	0.034	0.430**	0.025	− 0.134**
Grey matter volume	− 0.018	0.002	− 0.0001	0.006	− 0.026	− 0.675*	− 0.005	− 0.001
White matter volume	− 0.028	− 0.024	− 0.007	− 0.008	0.014	− 0.363*	0.099**	− 0.037
CSF volume	0.010	− 0.001	− 0.023	0.010	0.046*	0.526**	0.042**	0.014

** p-value ≤ 0.001; * p-value ≤ 0.01.

For the potential mediators, addiction, self-harm contemplation, and a higher neuroticism score significantly predicted more anxiety symptoms. Engaging in social activities was marginally associated with anxiety symptoms (β = − 0.015, *p* = .003), considering α = 0.001. No significant association was found between having been in a confiding relationship and anxiety symptoms (see Tables 4 and 5).

**Table 4:** Standardised coefficients of the model with coping strategies as predictors.

Outcomes	Coping strategies				
	Addiction	Social activities	Self-harm contemplation	Confiding relationship	Neuroticism
Depressive symptoms	0.065**	− 0.021**	0.112**	− 0.001	0.369**
Anxiety symptoms	0.049**	− 0.015*	0.074**	− 0.0004	0.422**
Fluid intelligence	0.019	0.025	0.006	0.088**	− 0.045**
Trail Making Test B	− 0.005	− 0.005	− 0.005	− 0.046**	0.078**
Grey matter volume	− 0.034	0.005	0.008	0.033	− 0.020
White matter volume	− 0.038	− 0.001	− 0.003	0.022	0.021
CSF volume	0.053**	− 0.007	0.002	− 0.011	0.002

** *p*-value ≤ 0.001, * *p*-value ≤ 0.01.

**Table 5:** Significant and marginal standardised estimates for the indirect associations tested.

Indirect effects	β
Addiction as mediator	
Sexual abuse ⇨ Addiction ⇨ Depressive symptoms	0.003**
Sexual abuse ⇨ Addiction ⇨ Anxiety symptoms	0.002**
Sexual abuse ⇨ Addiction ⇨ CSF volume	0.003**
Emotional neglect ⇨ Addiction ⇨ Depressive symptoms	0.002**
Emotional neglect ⇨ Addiction ⇨ Anxiety symptoms	0.002**
Emotional neglect ⇨ Addiction ⇨ CSF volume	0.002*
Physical abuse ⇨ Addiction ⇨ Depressive symptoms	0.001**
Physical abuse ⇨ Addiction ⇨ Anxiety symptoms	0.001**
Physical abuse ⇨ Addiction ⇨ CSF volume	0.001*
Emotional abuse ⇨ Addiction ⇨ Depressive symptoms	0.005**
Emotional abuse ⇨ Addiction ⇨ Anxiety symptoms	0.004**
Emotional abuse ⇨ Addiction ⇨ CSF volume	0.004**
Social activities as mediator	
Emotional neglect ⇨ Social activities ⇨ Depressive symptoms	0.001*
Self-harm contemplation as mediator	
Sexual abuse ⇨ Self-harm ⇨ Depressive symptoms	0.010**
Sexual abuse ⇨ Self-harm ⇨ Anxiety symptoms	0.007**
Emotional neglect ⇨ Self-harm ⇨ Depressive symptoms	0.008**
Emotional neglect ⇨ Self-harm ⇨ Anxiety symptoms	0.006**
Physical abuse ⇨ Self-harm ⇨ Depressive symptoms	0.006**
Physical abuse ⇨ Self-harm ⇨ Anxiety symptoms	0.004**
Emotional abuse ⇨ Self-harm ⇨ Depressive symptoms	0.015**
Emotional abuse ⇨ Self-harm ⇨ Anxiety symptoms	0.010**
Confiding relationship as mediator	
Physical neglect ⇨ Confiding relationship ⇨ fluid intelligence performance	− 0.009**
Physical neglect ⇨ Confiding relationship ⇨ TMTB	0.005**
Sexual abuse ⇨ Confiding relationship ⇨ fluid intelligence performance	0.002**
Sexual abuse ⇨ Confiding relationship ⇨ TMTB	− 0.001*
Emotional neglect ⇨ Confiding relationship ⇨ fluid intelligence performance	− 0.001**
Emotional neglect ⇨ Confiding relationship ⇨ TMTB	0.001*
Physical abuse ⇨ Confiding relationship ⇨ fluid intelligence performance	0.001**
Physical abuse ⇨ Confiding relationship ⇨ TMTB	− 0.001*
Neuroticism as mediator	
Physical neglect ⇨ Neuroticism ⇨ Depressive symptoms	0.004**
Physical neglect ⇨ Neuroticism ⇨ Anxiety symptoms	0.005**
Physical neglect ⇨ Neuroticism ⇨ TMTB	0.001**
Sexual abuse ⇨ Neuroticism ⇨ Depressive symptoms	0.011**
Sexual abuse ⇨ Neuroticism ⇨ Anxiety symptoms	0.013**
Sexual abuse ⇨ Neuroticism ⇨ fluid intelligence performance	− 0.001**
Sexual abuse ⇨ Neuroticism ⇨ TMTB	0.002**
Emotional neglect ⇨ Neuroticism ⇨ Depressive symptoms	0.038**
Emotional neglect ⇨ Neuroticism ⇨ Anxiety symptoms	0.043**
Emotional neglect ⇨ Neuroticism ⇨ fluid intelligence performance	-005**
Emotional neglect ⇨ Neuroticism ⇨ TMTB	0.008**
Emotional abuse ⇨ Neuroticism ⇨ Depressive symptoms	0.040**
Emotional abuse ⇨ Neuroticism ⇨ Anxiety symptoms	0.046**
Emotional abuse ⇨ Neuroticism ⇨ fluid intelligence performance	− 0.005**
Emotional abuse ⇨ Neuroticism ⇨ TMTB	0.009**

**p-value ≤ 0.001, *p-value ≤ 0.01.

### Depression

Sexual abuse, emotional neglect, physical abuse, and emotional abuse in childhood significantly predicted more depressive symptoms. No significant associations were found between physical neglect and depressive symptoms (see Table 3 and Supplementary Figure A).

For the potential mediators, addiction, self-harm contemplation, and a higher neuroticism score significantly predicted higher depressive symptoms, while engagement in social activities significantly predicted lower depressive symptoms. No significant association was found between having been in a confiding relationship and depressive symptoms (see Tables 4 and 5).

### Fluid intelligence

Physical neglect in childhood significantly predicted poorer fluid intelligence performance. No significant associations were found between sexual abuse, emotional neglect, physical abuse, emotional abuse, and performance in fluid intelligence (see Table 3 and Supplementary Figure A).

For the potential mediators, having been in a confiding relationship significantly predicted better fluid intelligence performance, while a higher neuroticism score significantly predicted poorer fluid intelligence performance. No significant associations were found between addiction, social activities, self-harm contemplation, and performance in fluid intelligence (see Tables 4 and 5).

### TMTB

Physical neglect in childhood significantly predicted slower TMTB completion time. No significant associations were found between sexual abuse, emotional neglect, physical abuse, emotional abuse, and TMTB completion time (see Table 3 and Supplementary Figure A).

For the potential mediators, having been in a confiding relationship significantly predicted faster TMTB completion time, while a higher neuroticism score significantly predicted slower TMTB completion time. No significant associations were found between addiction, social activities, self-harm contemplation, and TMTB completion time (see Tables 4 and 5).

### Grey matter

No significant associations were found between physical neglect, sexual abuse, emotional neglect, physical abuse, emotional abuse, and grey matter volume (see Table 3).

For the potential mediators, no significant associations were found between addiction, social activities, self-harm contemplation, having been in a confiding relationship, neuroticism score, and grey matter volume (see Tables 4 and 5).

### White matter

No significant associations were found between physical neglect, sexual abuse, emotional neglect, physical abuse, emotional abuse, and white matter volume (see Table 3).

For the potential mediators, no significant associations were found between addiction, social activities, self-harm contemplation, having been in a confiding relationship, neuroticism score, and white matter volume (see Tables 4 and 5).

### CSF

Emotional abuse in childhood was marginally associated with CSF volume (β = 0.046, *p* = .004), given the α = 0.001 threshold. No significant associations were found between physical neglect, sexual abuse, emotional neglect, physical abuse, and CSF volume (see Table 3 and Supplementary Figure A).

For the potential mediators, addiction significantly predicted a greater CSF volume. No significant associations were found between social activities, self-harm contemplation, having been in a confiding relationship, neuroticism score, and CSF volume (see Tables 4 and 5).

### Global addiction

Sexual abuse, emotional neglect, physical abuse, and emotional abuse significantly predicted having/having had an addiction. No significant associations were found between physical neglect and addiction (see Table 3 and Supplementary Figure A).

### Social activities

Emotional neglect in childhood significantly predicted a lower number of social activities undertaken. No significant associations were found between physical neglect, sexual abuse, physical abuse, and emotional abuse, and the number of social activities undertaken (see Table 3 and Supplementary Figure A).

### Self-harm

Sexual abuse, emotional neglect, physical abuse, and emotional abuse in childhood significantly predicted having contemplated self-harm. No significant associations were found between physical neglect and self-harm contemplation (see Table 3 and Supplementary Figure A).

### Confiding relationship

Physical neglect and emotional neglect in childhood significantly predicted not having been in a confiding relationship. Sexual abuse, physical abuse, and emotional abuse in childhood significantly predicted having been in a confiding relationship (see Table 3 and Supplementary Figure A).

### Neuroticism

Physical neglect, sexual abuse, emotional neglect, and emotional abuse in childhood significantly predicted a higher neuroticism score. No significant associations were found between physical abuse and neuroticism score (see Table 3 and Supplementary Figure A).

## Discussion

In this large population-based study, early adversity was consistently associated with adult anxiety symptoms, whereas associations with depressive symptoms, cognition, and brain measures varied by adversity type. By examining multiple adversities, coping strategies, neuroticism, and outcomes within a single integrative framework, the present findings highlight both the heterogeneity of adversity-related outcomes and the prominence of psychosocial pathways in explaining these associations.

All early adverse experiences were significantly associated with greater anxiety symptoms, in line with the previous literature^4,29^. In contrast, depressive symptoms were associated with all adversities except physical neglect, suggesting that different forms of adversity may confer differential risk for internalising outcomes. These findings reinforce the importance of distinguishing between adversity types rather than treating early adversity as a unitary exposure.

Notably, neglect-related adversities showed distinct patterns in social and cognitive domains. Emotional and physical neglect were negatively associated with being in a confiding relationship, and emotional neglect was associated with lower participation in social activities. These findings suggest that neglect, particularly emotional neglect, may exert a lasting impact on social functioning, potentially reflecting early deprivation of relational and emotional resources. This interpretation aligns with the threat–deprivation framework, which proposes that deprivation-related adversities preferentially affect cognitive and social development, whereas threat-related adversities more strongly influence emotional processing^30,31^.

Physical neglect was the only adversity directly associated with poorer cognitive performance, as indicated by lower fluid intelligence scores and slower Trail Making Test B completion times. This contrasts with previous results that also identified sexual abuse as a predictor of poorer cognition^7^. In the present model, associations between other adversities and cognition were fully accounted for by indirect pathways involving neuroticism and confiding relationships. This suggests that, for several adversity types, cognitive outcomes may be more closely linked to enduring personality traits and social functioning than to direct effects of early adversity.

Maladaptive behavioural responses to stress (i.e., addiction and self-harm contemplation) were associated with all adversity types except physical neglect, and with both anxiety and depressive symptoms. This finding is consistent with previously published studies linking early adversity with substance use and self-harm-related behaviours^14,29,32–34^. The absence of a significant association between physical neglect, addiction, self-harm contemplation, and depressive symptoms may reflect mechanism(s) specific to physical neglect (e.g., financial resources) that differ from those underlying emotional neglect (e.g., psychological and emotional resources promoting resilience^35–37^. Importantly, the lack of a direct association between physical neglect and depressive symptoms may be explained by the significant indirect association operating through neuroticism.

Neuroticism emerged as a particularly prominent correlate, mediating associations between multiple adversity types and mental health outcomes, as well as cognitive performance. All forms of adversity, except physical abuse, were significantly associated with higher neuroticism scores, consistent with evidence that early stress contributes to negative emotionality and stress sensitivity^13,22^. Given the strong associations between neuroticism and both anxiety and depression, these findings suggest that trait vulnerability may account for a substantial proportion of adversity-related mental health risk in adulthood.

In contrast to expectations, we found no relationship between early adversity and global grey- or white-matter volumes. These null results are noteworthy, particularly in light of prior reports of structural brain differences associated with early adversity^38^. The null findings may be explained by the substantially smaller imaging subsample for grey-matter, white-matter, and CSF volume measures, which likely reduced statistical power to detect small effect sizes typically observed in population-based neuroimaging studies. It is also possible that global volumetric measures are relatively insensitive to adversity-related effects in mid-to-late adulthood, or that age- and sex-related variance accounts for a substantial proportion of variability in these measures. Alternatively, previously reported associations may reflect region-specific effects or sample-specific biases that do not generalise to large, population-based cohorts. The limited and indirect associations observed with CSF volume further suggest that structural brain alterations may not constitute a primary pathway linking early adversity to adult mental health outcomes at the level of global brain metrics.

Taken together, these findings underscore the importance of adversity specificity and suggest that psychosocial and personality-related pathways may be more robustly associated with adult mental health and cognition than global brain structure^11^. Although numerous indirect associations reached statistical significance, effect sizes were generally small, likely reflecting the large sample size rather than strong mechanistic effects. As such, the present results should be interpreted as mapping patterns of association rather than identifying causal mechanisms.

## Strengths and limitations

A major strength of this study is the integration of multiple forms of early adversities, coping strategies, personality traits, and adult outcomes in one analytical framework, allowing a broad examination of how these variables relate to one another. The large sample size provides high statistical power and enables the detection of robust patterns of association.

Several limitations should be acknowledged. Although taken into account in our analysis, the large sample comprises missing values, including on the early adversity items (from 67.85% to 68.15% of missing values). The complexity of the model and the large sample size increase the likelihood of statistically significant paths with small effect sizes, necessitating cautious interpretation. Applying a stringent significance threshold (α = 0.001) helped limit type I errors, although this inevitably increased the risk of type II errors. The generalisability of the results is limited by the unbalanced sex distribution of the sample size and by the analyses being restricted to participants of White ethnicity. Sex-specific and ethnicity-related effects remain important avenues for future research, particularly in samples with sufficient size to support adequately powered stratified analyses. This research used cross-sectional data; however, future studies should use longitudinal data to obtain information on temporality. Another limitation is the use of self-reported retrospective early adversity items, which may be subject to several biases (e.g., survival, selection, and resilience bias, recall, social and mental health bias)^39^. However, the reliability of self-reported adversity has already been emphasised and might be even under-reported^40–42^. In addition, future studies need to focus on the role of genetics as well as learning behaviour in the adoption of coping strategies. Finally, it would be important to further investigate the social component involved in the experience of neglect as well.

## Conclusion

In this large population-based study, early adversity was robustly associated with adult anxiety symptoms, whereas associations with depressive symptoms, cognition, and brain measures varied by adversity type. Distinct patterns were observed for neglect-related adversities, which were particularly linked to social functioning and cognitive outcomes. Coping strategies and neuroticism accounted for several associations between early adversity and adult outcomes, highlighting the relevance of psychosocial and personality-related pathways. However, effect sizes were small and global brain volumes showed limited associations, underscoring the need for cautious interpretation. These findings suggest that considering adversity specificity and broader psychosocial context may be more informative than focusing on single pathways when studying the long-term correlates of early adversity.

## Supplementary Information

Below is the link to the electronic supplementary material.

Supplementary Material 1

## Data Availability

The data used in this study came from the UK Biobank ( [http://www.ukbiobank.ac.uk](http:/www.ukbiobank.ac.uk) ) application number 15697 (PI John Gallacher) and can be requested through the UK Biobank website ( [https://www.ukbiobank.ac.uk/enable-your-research/apply-for-access](https:/www.ukbiobank.ac.uk/enable-your-research/apply-for-access).) ).
