# Supplementary material for: Associations between early-life adversity, coping strategies, and adult mental health, brain, and cognition
Source: Sci Rep. 2026 Mar 4;16:12147. doi: 10.1038/s41598-026-42435-w (PMC13076903; doi:10.1038/s41598-026-42435-w)

**Figure A**

*Visual Summary of the Results found between Early Adversity and the Outcomes of Interest*


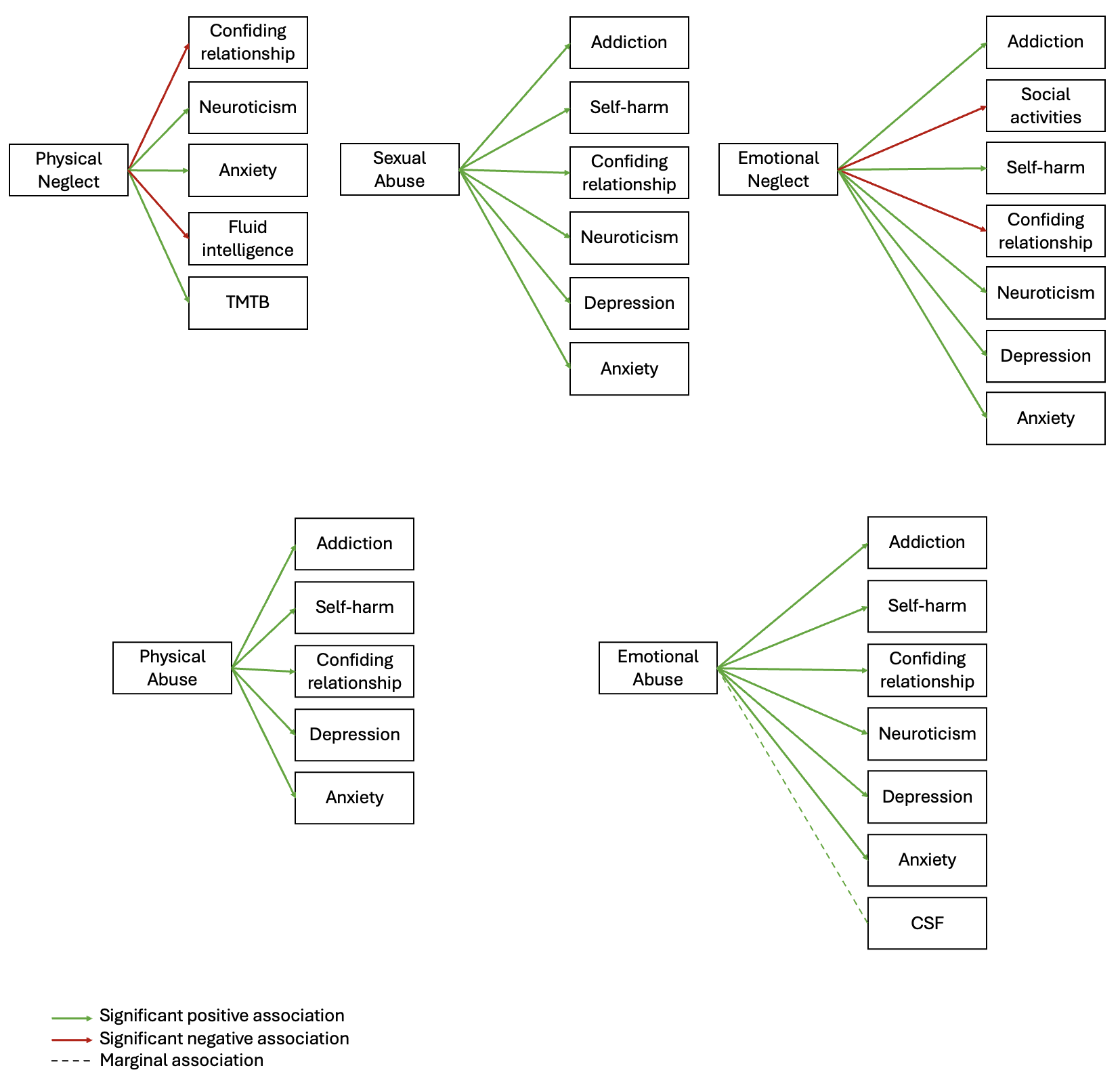

Supplement: Supplementary file 1 — Supplementary Material 1 [file 41598_2026_42435_MOESM1_ESM.docx]
